# The effects of electroporation buffer composition on cell viability and electro-transfection efficiency

**DOI:** 10.1038/s41598-020-59790-x

**Published:** 2020-02-20

**Authors:** Joseph J. Sherba, Stephen Hogquist, Hao Lin, Jerry W. Shan, David I. Shreiber, Jeffrey D. Zahn

**Affiliations:** 1 Rutgers, The State University of New Jersey, Department of Biomedical Engineering, Piscataway, 08854 United States; 2 Rutgers, The State University of New Jersey, Department of Mechanical and Aerospace Engineering, Piscataway, 08854 United States

**Keywords:** Transfection, Transfection

## Abstract

Electroporation is an electro-physical, non-viral approach to perform DNA, RNA, and protein transfections of cells. Upon application of an electric field, the cell membrane is compromised, allowing the delivery of exogenous materials into cells. Cell viability and electro-transfection efficiency (eTE) are dependent on various experimental factors, including pulse waveform, vector concentration, cell type/density, and electroporation buffer properties. In this work, the effects of buffer composition on cell viability and eTE were systematically explored for plasmid DNA encoding green fluorescent protein following electroporation of 3T3 fibroblasts. A HEPES-based buffer was used in conjunction with various salts and sugars to modulate conductivity and osmolality, respectively. Pulse applications were chosen to maintain constant applied electrical energy (J) or total charge flux (C/m^2^). The energy of the pulse application primarily dictated cell viability, with Mg^2+^-based buffers expanding the reversible electroporation range. The enhancement of viability with Mg^2+^-based buffers led to the hypothesis that this enhancement is due to ATPase activation via re-establishing ionic homeostasis. We show preliminary evidence for this mechanism by demonstrating that the enhanced viability is eliminated by introducing lidocaine, an ATPase inhibitor. However, Mg^2+^ also hinders eTE compared to K^+^-based buffers. Collectively, the results demonstrate that the rational selection of pulsing conditions and buffer compositions are critical for the design of electroporation protocols to maximize viability and eTE.

## Introduction

The ability to perform DNA, RNA, and protein transfection in a safe and efficient manner is increasingly important in both biomedical and clinical research^1–3^. Currently, the gold standard for gene delivery is the use of viruses to perform DNA transfection. Though viral-mediated gene delivery has been shown to be effective, as demonstrated through the recent FDA approval of initial cell therapies, this delivery modality suffers from several drawbacks^4^. The problems associated with viral transfection include cost, cytotoxicity, immunogenicity, mutagenesis/tumorigenesis potential, and size capacity restrictions on the gene to be delivered^5–7^. These disadvantages have led to the continued development of non-viral alternatives.

Over the last 40 years, electroporation has emerged as an attractive approach for delivery of exogenous materials into cells and tissues. Electroporation is a non-viral technique used to deliver DNA, RNA, and proteins (including plasmid DNA (pDNA) vectors) to biological cells. Through the application of external electric fields of appropriate strength, duration, form, and number, a reversible increase in permeability is achieved to allow delivery of both small and large molecules through an otherwise impermeable cell membrane^8^. For many applications, electroporation is advantageous compared to viral-mediated gene delivery. When applied appropriately, it is generally inexpensive, safe, easy to operate, and efficient in performing transfections of cells from a variety of lineages^9^. However, when not optimized, electroporation can induce significant cell death from excessive permeabilization of a cell or generate insufficient transfection efficiency when permeabilization is limited.

Electroporation outcomes are typically defined as the resulting cell viability, defined as the percentage of living cells following electroporation compared to a non-electroporated control, and electro-transfection efficiency (eTE), defined as the percentage of cells receiving or expressing the delivered vector. These outcomes are dependent on a variety of experimental parameters including: electric pulse strength and duration, number of electric pulses applied, cell type, cell density, pDNA concentration, buffer conductivity, and buffer composition^8–12^. Not only does such a large number of experimental variables increase the complexity of protocol optimization, it has led to a vast landscape of published work, making it difficult to draw conclusions among them. This has given rise to numerous electroporation protocols and electroporation buffers used across laboratories or commercial offerings, and it can often be unclear why a particular buffer was selected for a given cell type, application, or protocol. Electroporation buffers generally fall into several categories of composition – saline-based, phosphate-based, HEPES-based, or cell-culture-media based – with conductivity tailored by the salt added and osmolality adjusted with an osmotic agent, often sugar or an inert protein^13–15^.

The effect of electroporation buffer composition on propidium iodide (PI) uptake into myeloma cells has been previously been investigated^10^. In this study, electroporation buffers of various conductivities were made using K^+^, Na^+^, C1^−^ and SO_4_^2−^ as ions. Following electroporation, PI uptake into the myeloma cells was not significantly different regardless of ionic composition at a fixed medium conductivity. However, medium conductivity did affect viability, with low conductivity buffers of the same ionic composition producing lower viability following electroporation. There were also differences in viability between Na^+^ and K^+^ based buffers at higher field strengths (>5 kV/cm) but similar viabilities at lower field strengths. These early studies motivated us to explore how buffer composition can affect electroporation outcomes.

Recently, we have explored how pulsing conditions affect the delivery of small, membrane impermeant dyes such as PI and larger macromolecules such as fluorescein-conjugated dextrans, as well as the effects on the short-term (<2 hours) viability of cells following electroporation^16–20^. For these studies we used a low conductivity HEPES-based buffer with the electrolyte conductivity tailored via the addition of MgCl_2_. This buffer composition exploited an electrokinetic phenomena known as Field Amplified Sample Stacking (FASS), which exploits conductivity differences between the intracellular and extracellular environments, resulting in an increase in small molecule delivery to the cytosol following electroporation^18,19^. In a subsequent study, we investigated a two-pulse electroporation protocol, consisting of a high intensity, short duration electric pulse to permeabilize the cells, followed by a lower intensity, long duration electric pulse to enhance delivery of exogenous materials into the cytoplasm^21^. One motivating observation from this work was a strong, negative correlation between short term cell viability and the total applied electrical energy of the second applied pulse in all experiments conducted. However, in this earlier work, different HEPES-based electroporation buffer conductivities were tested, but only one salt, MgCl_2_, was used to titrate the buffer to the desired conductivity.

In this paper, we expand our investigation to explore different electroporation buffer compositions, showcasing the effect of different salts and sugars to study the effects of applied electrical energy on long-term cell viability and eTE of pDNA vectors. We systematically explore different buffer compositions and their effect on the electroporation outcomes of cell viability and transfection efficiency for pDNA encoding green fluorescent protein (GFP) 24 hours following electroporation. We also pre-determined the electrical pulsing parameters to keep either the total applied electrical energy (J) or the total ionic charge movement per unit area, i.e. charge flux (C/m^2^), a constant. The resulting cell viability was found to be dependent on the applied pulse applied electrical energy, with different buffer compositions expanding the reversible electroporation capabilities of the cell populations. In particular, the presence of Mg^2+^ ions enhanced the ability of cells to recover following high-energy pulse applications. This led us to hypothesize that the effect of magnesium on post electroporation viability is due to magnesium’s role in the activation of the ATPase membrane ion channels. Preliminary supporting evidence for this mechanism is shown by inhibiting these enzymes during electroporation with the addition of an ATPase inhibitor, lidocaine. However, Mg^2+^ concentrations need to be optimized, as the ion also hinders eTE when compared to K^+^-based buffers. The approach established and presented in this study allows for a better understanding of the effects that different electroporation buffer solutions have on electroporation outcomes and highlights the importance of buffer recipe in the optimization of electroporation protocols.

## Materials and Methods

### Cell culture

NIH-3T3 mouse fibroblasts were cultured in DMEM supplemented with 10% v/v fetal bovine serum, 1% v/v L-glutamine, and 1% penicillin-streptomycin (Sigma-Aldrich, St. Louis, MO). Cells were plated at a cell density of 1.65 × 10^5^ cells/mL for 24 hours at 37 °C, in a 95% O_2_/5% CO_2_ incubator (Thermo Electron Corp., Marietta, OH) prior to electroporation experiments.

### Electroporation buffer preparation

A HEPES-based electroporation buffer was used for the current study. Buffer pH was titrated to 7.4 using NaOH. Buffer osmolality was balanced to ~300 mOsm using a commercial osmometer (model 3D3, Advanced Instruments, Norwood, MA) with either sucrose or trehalose as the osmotic balancing agent. Buffer conductivity was adjusted to either 500 μS/cm or 2000 μS/cm using a variety of salts: MgCl_2_, KCl, MgSO_4_, or a MgCl_2_/KCl mixture. Buffer conductivity was measured using a commercial conductivity meter (model COND 6+ , Oakton Instruments, Vernon Hills, IL). Table 1 lists detailed information regarding the electroporation buffers tested in this study. For lidocaine experiments, lidocaine hydrochloride was diluted to a final concentration of 10 mM within the cell resuspension. All molecular additives were purchased from Sigma-Aldrich (Sigma-Aldrich, St. Louis, MO).

**Table 1:** Electroporation Buffer Concentrations.

Salt	Concentration (mM)	Sugar	Conductivity (μS/cm)
MgCl_2_	1.5	Sucrose	500
MgSO_4_	1.8	Sucrose	500
MgCl_2_/KCl	0.7/1	Sucrose	500
KCl	2	Sucrose	500
KCl	2	Trehalose	500
MgCl_2_	10	Sucrose	2000
KCl	10	Sucrose	2000

All buffers contain 10mM HEPES and 3mM NaOH. Both sucrose and trehalose are at a final concentration of 285 mM for all buffers.

### DNA plasmid

Plasmid pMAX-GFP (Lonza, Walkersville, MD) was procured at a concentration of 1 μg/μL from commercially available cell transfection kits (Lonza, Cat No.:VCA-1003, Lot F-12559, endotoxin levels <1 pg/μg plasmid). Plasmid vectors were loaded at a final concentration of 20 μg/mL prior to electroporation^22–24^.

### Electroporation pulse parameters

A square wave generator, BTX ECM 830 (Harvard Bioscience Inc., Holliston, MA), was used to generate a single electrical pulse of pre-determined electric-field strength and duration to an electroporation cuvette. Electric field strength was nominally calculated as: *E* = *V*/*d*, where *E* is the electric field strength (kV/cm), *V* is the applied voltage (kV), and *d* is the distance between the electrodes (0.2 cm) in the cuvette. A 1.2 kV/cm pulse for 1 ms in duration was used as the control pulse for determining the remaining pulses in the study. Pulse applications were chosen to conserve either the total applied electrical energy (*W* = *σ* × *E*^2^ × *t* × *υ*) or total charge flux (*Φ*_Q_ = *σ* × *E* × *t*), where *W* is the total applied electrical energy (J), *σ* is the electroporation buffer conductivity (S/m), *E* is the applied electric field (V/m), *t* is the pulse duration (s), *υ* is the total electroporation buffer volume (m^3^), and *Φ*_Q_ is the total charge flux (C/m^2^). The pulsing conditions applied for constant total applied energy and constant total charge flux can be found in Tables 2 and 3, respectively. For each pulsing condition used in this study, temperature changes due to Joule heating of the electrolyte were conservatively calculated assuming all the electrical energy, *W*, is converted to heat in the solution as *W = ρ* × *υ* × *C*_*p*_ × *ΔT*, where *ρ* is the solution density (1,000 kg/m^3^), *υ* is the cuvette buffer volume (1 × 10^−7^ m^3^), *C*_*p*_ is the heat capacity of water at room temperature (4,184 J/kgC), and *ΔT* is the temperature change from the electroporation pulse. From these calculations, the temperature change from Joule heating is less than 0.75 °C and 3 °C for the 500 μS/cm and 2000 μS/cm buffers, respectively, for all pulse conditions tested and are considered negligible in this study.

**Table 2:** Electroporation outcomes for constant applied energy.

		Sucrose, MgCl_2_ (2000 μS/cm)		Sucrose, KCl (2000 μS/cm)		Sucrose, MgCl_2_ (500 μS/cm)		Sucrose, MgSO_4_ (500 μS/cm)		Sucrose, MgCl_2_/KCl (500 μS/cm)		Sucrose, KCl (500 μS/cm)		Trehalose, KCl (500 μS/cm)	
Pulse Strength (kV/cm)	Pulse Duration (ms)	Via. (%)	eTE (%)	Via. (%)	eTE (%)	Via. (%)	eTE (%)	Via. (%)	eTE (%)	Via. (%)	eTE (%)	Via. (%)	eTE (%)	Via. (%)	eTE (%)
1.2	1.00	77 ± 19	11 ± 3.0	79 ± 4.6	21 ± 3.2	91 ± 2.6	12 ± 4.0	88 ± 8.7	14 ± 3.8	90 ± 26	29 ± 19	101 ± 22	32 ± 13	68 ± 17	36 ± 7.8
1.8	0.44	86 ± 9.5	8 ± 0.6	77 ± 9.9	17 ± 6.1	96 ± 3.5	8 ± 1.2	96 ± 42	16 ± 5.6	88 ± 20	24 ± 6.2	72 ± 7.8	22 ± 1.5	53 ± 18	43 ± 3.2
2.4	0.25	84 ± 8.1	14 ± 1.5	69 ± 13	28 ± 3.1	87 ± 5.9	17 ± 3.5	76 ± 3.1	21 ± 9.3	94 ± 17	35 ± 16	83 ± 18	36 ± 11	38 ± 4.6	48 ± 18
3.6	0.11	65 ± 6.2	19 ± 7.8	63 ± 9.3	23 ± 4.6	95 ± 10	13 ± 1.0	84 ± 21	21 ± 5.1	83 ± 8.6	27 ± 2.9	56 ± 7.0	31 ± 12	34 ± 12	49 ± 15
4.8	0.06	58 ± 8.0	30 ± 2.5	52 ± 20	37 ± 7.0	78 ± 13	22 ± 4.0	81 ± 6.2	23 ± 11	88 ± 4.7	34 ± 12	63 ± 20	47 ± 11	27 ± 4.5	52 ± 13

**Table 3:** Electroporation outcomes for constant charge flux.

		Sucrose, MgCl_2_ (2000 μS/cm)		Sucrose, KCl (2000 μS/cm)		Sucrose, MgCl_2_ (500 μS/cm)		Sucrose, MgSO_4_ (500 μS/cm)		Sucrose, MgCl_2_/KCl (500 μS/cm)		Sucrose, KCl (500 μS/cm)		Trehalose, KCl (500 μS/cm)	
Pulse Strength (kV/cm)	Pulse Duration (ms)	Via. (%)	eTE (%)	Via. (%)	eTE (%)	Via. (%)	eTE (%)	Via. (%)	eTE (%)	Via. (%)	eTE (%)	Via. (%)	eTE (%)	Via. (%)	eTE (%)
1.2	1.00	77 ± 19	11 ± 3.0	79 ± 4.6	21 ± 3.2	91 ± 2.6	12 ± 4.0	88 ± 8.7	14 ± 3.8	90 ± 26	29 ± 19	101 ± 22	32 ± 13	68 ± 17	36 ± 7.8
1.8	0.67	80 ± 4.7	11 ± 0.6	44 ± 12	32 ± 5.5	91 ± 5.1	9 ± 1.0	102 ± 5	15 ± 4.2	88 ± 3.6	20 ± 4.6	49 ± 6.1	40 ± 4.2	30 ± 11	48 ± 2.9
2.4	0.50	50 ± 6.0	34 ± 1.5	24 ± 4.0	57 ± 5.8	82 ± 9.1	26 ± 3.5	79 ± 14	25 ± 8.6	87 ± 8.5	42 ± 14	30 ± 10	73 ± 8.7	21 ± 9.5	67 ± 21
3.6	0.33	24 ± 5.9	55 ± 6.7	11 ± 5.9	57 ± 2.5	83 ± 7.0	18 ± 1.5	80 ± 26	21 ± 4.4	67 ± 14	42 ± 3.8	6.3 ± 2.5	71 ± 2.6	24 ± 17	52 ± 17
4.8	0.25	18 ± 4.0	65 ± 7.6	7 ± 2.1	80 ± 10	61 ± 12	43 ± 12	49 ± 4.0	42 ± 16	39 ± 11	70 ± 9.8	9.0 ± 4.4	82 ± 1.0	8 ± 4.4	69 ± 24

### Cell harvest and electroporation

3T3 fibroblast cells were harvested for experiments 24 hours following cell passage. An electroporation protocol was adapted from *Potter & Heller* and our previous work^13,16–21^. Briefly, following trypsinization, cells were resuspended in antibiotic-free media and centrifuged for 2 minutes at 2000 rpm. The cells were washed using the electroporation buffer under investigation. They were then resuspended at a concentration of 3 × 10^6^ cells/mL in a 0.2 cm gap electroporation cuvette (Fisher Scientific, Waltham, MA), which included the pMAX GFP vector at a final concentration of 20 μg/mL. The total resuspension volume was 100 μL. The cuvettes were then placed on ice for 10 minutes prior to pulse application. Control experiments were conducted for each individual experiment for which the entire experimental procedure was followed but no electrical pulse was delivered. The exterior of the cuvette electrodes were dried, and the cuvettes were secured in the BTX cuvette safety stand where electrical contact was verified with a multimeter. Pulses were applied at room temperature in sterile fashion. Following pulse application, cuvettes were briefly placed on ice before the cells were transferred to a pre-warmed (37 °C) tissue culture plate containing antibiotic free media and incubated for 24 hours prior to imaging. Cuvettes were discarded after a single use.

### Cell viability and gene electro-transfection efficiency

Quantification of viability and eTE used a protocol adapted from Haberl *et al*.^23^ Following 24 hours of incubation, cells were washed with PBS and then imaged under phase contrast and epifluorescence microscopy (FITC filter) using a 10× objective to determine the resulting cell viability and eTE, respectively (Microscope: Olympus IX81, Japan, Camera: Hamamatsu Photonics, Model: C4742-95-12G04, Japan, Software: MetaMorph). Images were captured from 5 random locations to gather representative images of the overall population for each experimental condition. Cell viability was determined by normalizing the total cell count per experimental condition to the total cell count in the no pulse control condition. Gene eTE was defined as the ratio of the total number of GFP-positive cells to the total number of viable cells per experimental condition.

### Statistical analysis

All experiments were independently run in triplicate (n = 3) with the results represented as mean ± standard deviation. Results were analyzed using a two-way ANOVA followed by a Tukey multiple comparison test (GraphPad Prism v7, GraphPad Software, La Jolla, CA) with *p* < 0.05 considered statistically significant. Results from the two-way ANOVAs and statistically significant results from the multiple comparison tests can be found in Supplementary Tables 1 and 2, respectively.

## Results and Discussion

The purpose of this study was to distinguish the effects of different buffer solutions and pulse characteristics on electroporation outcomes. Tables 2 and 3 display the viability and eTE results gathered from the constant-applied-energy and constant-total-charge-flux pulse applications, respectively, for all electroporation buffers tested in the study.

### Cell viability and electro-transfection efficiency: constant applied electrical energy

The effects of buffer composition and charge flux on cell viability and eTE were evaluated in conditions where applied energy was held constant (Table 2). Separate two-way ANOVAs were performed for two different conductivities. Plots of viability for both conductivities are found in Supplementary Fig. 1. For the 500 μS/cm buffers, cell viability was not significantly affected by buffer composition, the charge flux, or the interaction between these. For buffers with 2000 μS/cm conductivity, the effects of charge flux on viability are significant (*p* = 0.0044), whereas the buffer composition and the interaction between the two variables do not have a significant effect. Together, these results generally support our previous observations that cell viability following electroporation is largely dependent on the overall electrical energy applied during transfection procedures^20^.

When analyzing the effects that buffer composition, charge flux, and the interaction of these variables have on the eTE, a similar trend is found (refer to Table 2 for eTE values). A two-way ANOVA of the results with 500 μS/cm and with the 2,000 μS/cm conductivity buffers indicated that both buffer composition (*p* < 0.0001) and charge flux (*p* = 0.0073/*p* < 0.0001) have significant effects on eTE, and that the interaction of these variables is not significant. However, the significant effect of buffer composition on eTE results from the different molecular contents (i.e., Mg^2+^ vs. K^+^-based buffers) of the electroporation buffer. This is better understood when comparing electroporation outcomes for different energy pulse applications.

### Cell viability and electro-transfection efficiency: constant charge flux

**Figure 1:**
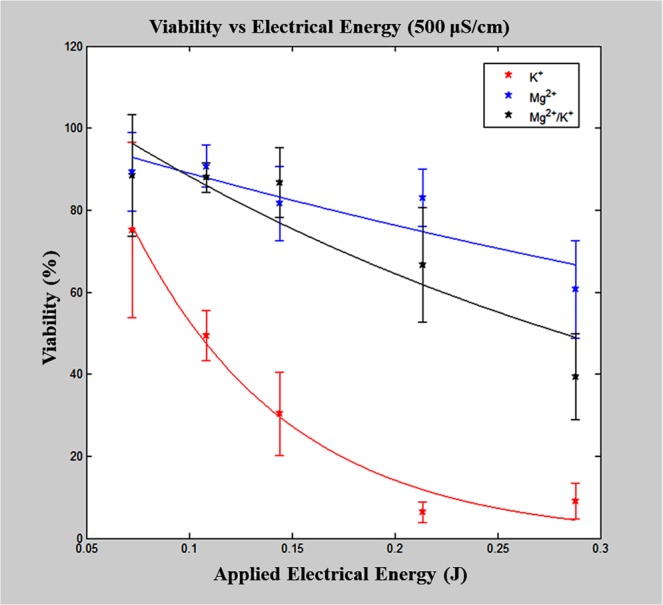
Viability versus applied electrical energy. Each buffer with a final conductivity of 500 μS/cm with Cl^−^ as the anion and sucrose as the osmotic balancing agent. Mg^2+^ and Mg^2+^/K^+^-buffers had significantly greater viability results (*p* < 0.05) when compared to the K^+^-buffer. Mg^2+^-containing buffers resulted in a linear viability response, whereas the K^+^-buffer resulted in an exponential decay viability response curve.

The effects of buffer composition and applied electric pulse energy on cell viability and eTE were examined with conditions where charge flux was held constant (Table 3). Figure 1 is a plot of cell viability versus applied electrical energy for the constant charge flux experiments using the Mg^2+^ and K^+^-based buffer solutions at a final conductivity of 500 μS/cm. Two separate two-way ANOVAs were performed to examine the effects buffer composition and applied pulse energy have on cell viability. The first analysis includes all buffer compositions at 500 μS/cm. The analysis showed that the buffer composition, pulse energy, and the ordinal interaction between the two all had significant effects on cell viability (*p* < 0.0001). However, a noticeable difference in the viability versus applied energy curve for different buffer compositions is shown in Fig. 1, with two distinct cell viability responses observed, i.e. linear versus nonlinear decay, for the Mg^2+^-containing and Mg^2+^-lacking buffer compositions, respectively. A two-way ANOVA of results with only the Mg^2+^-containing buffer compositions (MgCl_2_, MgCl_2_/KCl, MgSO_4_) indicated that the energy of the pulse application has a significant effect on cell viability (*p* < 0.0001), while both buffer composition and the interaction between variables is not significant, with the post hoc analysis resulting in no statistical significance achieved at any pulse condition between buffer compositions. A visual representation of this can be found in Supplementary Fig. 2. This demonstrates the importance of having Mg^2+^ in the buffer composition for cell viability, enhancing the range of reversible electroporation for cell populations. These results are in agreement with other reports, indicating that Mg^2+^ is essential in preserving cell viability^22,23^.

**Figure 2:**
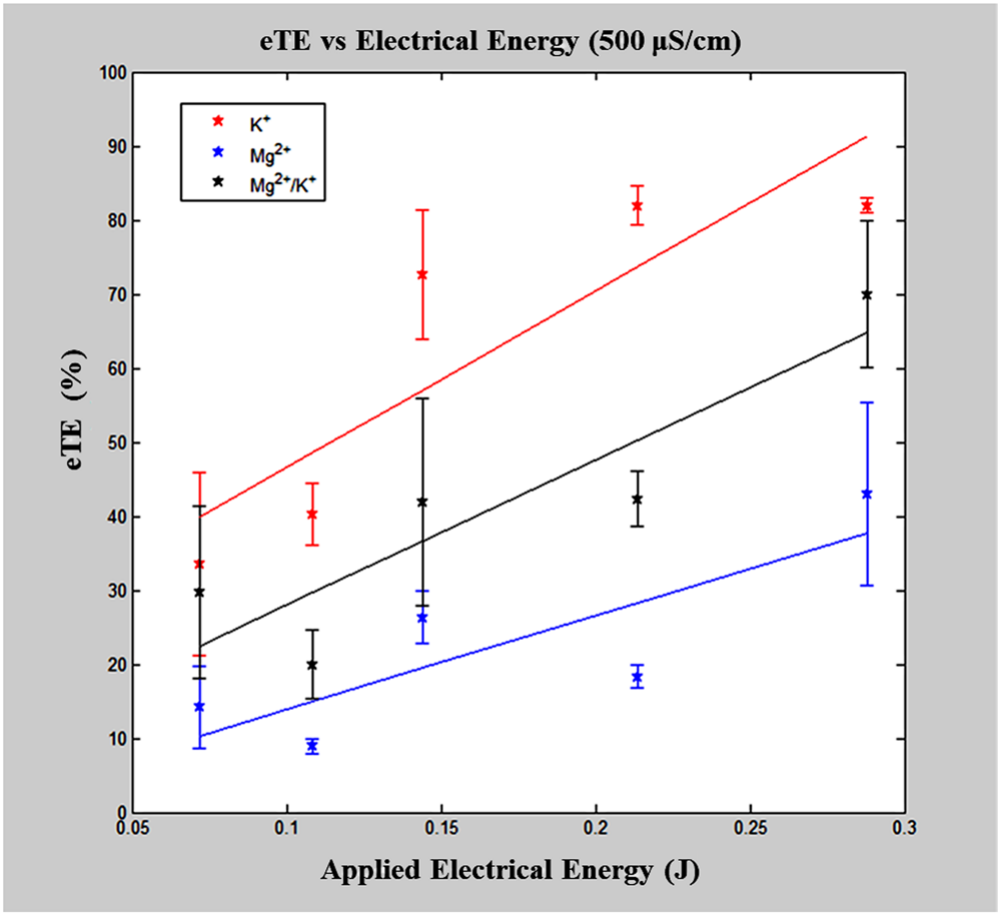
Electro-transfection efficiency versus applied electrical energy. Each buffer with a final conductivity of 500 μS/cm with Cl^−^ as the anion and sucrose as the osmotic balancing agent. The presence of Mg^2+^ leads to lower levels of eTE compared to the KCl-based buffer, with higher concentrations of Mg^2+^ further enhancing this observed effect. A linear increase in eTE was observed with increasing applied energy in all buffer compositions.

Although Mg^2+^ ions appear to preserve cell viability, the addition of magnesium can dramatically reduce the eTE^25,26^. When comparing results for the eTE, Mg^2+^-containing buffers result in lower eTEs compared to that of the K^+^-based buffer solutions (Fig. 2). In a similar fashion, two separate two-way ANOVA were performed to analyze the 500 μS/cm data (Mg^2+^-containing buffers and all buffers). The two-way ANOVA results when comparing all buffer solutions show that pulse energy (*p* < 0.0001), buffer composition (*p* < 0.0001), and the interaction between variables (*p* = 0.148, ordinal) all have significant effects on eTE. A separate analysis of only Mg^2+^-containing buffer compositions indicates that the applied pulse energy and the buffer composition significantly effect eTE (*p* < 0.0001), but the interaction between them is not significant. Post hoc comparisons revealed that higher concentrations of Mg^2+^ negatively impact eTE. In particular, the combination buffer of MgCl_2_/KCl resulted in significantly greater eTEs when compared against buffers containing only MgCl_2_ and MgSO_4_. Altogether, these results suggest an optimal concentration of Mg^2+^ could be determined for an electroporation buffer, as the Mg^2+^ ion plays a major and competing role in cell viability and transfection efficiency.

### Role of magnesium during electroporation

These findings led us to hypothesize that effects of Mg^2+^ on viability and eTE are due to the interaction between Mg^2+^ and nucleic acids, the role that Mg^2+^ plays as a co-factor for biochemical reactions, or a combination of the two. Mg^2+^ is an essential divalent cation that is required for the activation of numerous cellular enzymes.

Using fluorescently labeled pDNA vectors, Haberl *et al*. reported an enhanced interaction between the cell membrane and pDNA with increasing concentrations of Mg^2+^^22^. Although they believe this to be a necessary step for transfection, it is possible this interaction leads to lower eTE as more DNA is captured on the membrane surface and fewer vectors permeate through the membrane into the intracellular space. Another mechanism that may lead to lower eTE is the role of the magnesium ion as a co-factor for biochemical reactions, in particular the activation of DNase and RNase enzymes, leading to the degradation of the pDNA or translated mRNA prior to protein synthesis^27^. This hypothesis has been tested previously via non-specific enzyme inhibition using Zn^2+^^28^. Delgado-Canedo *et al*. examined the effect of adding Zn^2+^ to the buffer solution either prior to electroporation, during electroporation, or immediately following electroporation. They reported a 12% enhancement in eTE when Zn^2+^ was added immediately following electroporation compared to no Zn^2+^ added^29^. This methodology was repeated by Haberl *et al*., but no changes in eTE with the presence of Zn^2+^ were reported^23^. We also repeated this experiment, and found no increase in eTE in any of the three Zn^2+^ application conditions (data not shown). The inability to reproduce this experiment is likely the result of experimental variability, as the inhibition is dependent on the timing of Zn^2+^ application. However, the use of Zn^2+^ resulted in a decrease in cell viability presumably due to the inhibition of membrane-protein ion channels.

### ATPase inhibition

Upon exposure to the high-intensity external electric field during electroporation, intracellular ionic homeostasis is disturbed, with the resulting cell viability dependent on the recovery of this homeostatic environment^30^, presumably through the sodium-potassium ATPase ion pump. Rols *et al*. demonstrated that depleting ATP in CHO cells via incubation in sodium azide and 2-deoxy-D-glucose did not affect permeabilization efficiency but had a dramatic effect on viability following electroporation^31^_._ In addition to the activation of DNase/RNase enzymes, Mg^2+^ is also required for the activation of ATPase ion transporters^27,32^. Pilotelle-Bunner *et al*. reported that Mg^2+^ has a high binding affinity (*K*_*d*_ = 0.069 mM) for this enzyme, with enzyme activity saturating at ~ 1 mM Mg^2+^. These protein transporters are responsible for the active transport of critical ions (Na^+^, K^+^, Cl^−^, etc.) across the cell membrane^32^. Hence, the presence of Mg^2+^ in electroporation buffers may enhance cell viability by accelerating the re-establishment of ionic homeostasis, even at higher-energy pulse applications.

**Figure 3:**
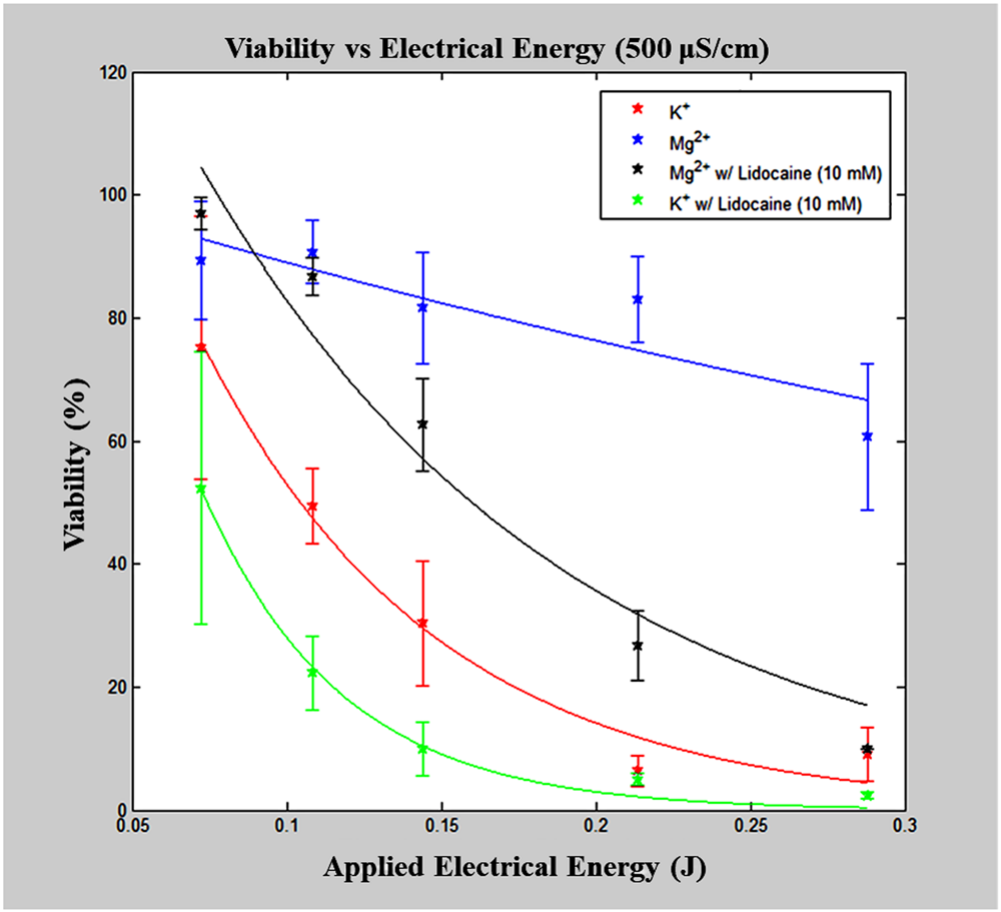
Membrane ATPase inhibition. Cell viability was assessed for both MgCl_2_ and KCl-buffers at 500 μS/cm with the addition of lidocaine, an ion channel inhibitor, at a final concentration of 10 mM. A significant difference is found in resulting cell viability when lidocaine is present even in the presence of Mg^2+^, most notably the shift in the viability response curve resembling that of the KCl-based buffer composition.

To preliminarily examine this hypothesis, lidocaine, a known ATPase ion channel inhibitor, was added to the electroporation buffer solutions (KCl and MgCl_2_ at 500 μS/cm) at a final concentration of 10 mM^33–35^. Electric pulses of different applied energy were delivered, with cell viability assessed at 24 hours. Figure 3 is a plot of cell viability versus applied electrical energy for both buffers with and without the addition of lidocaine. Both buffer composition (i.e., presence of lidocaine) and pulse energy significantly affected cell viability. (Please refer to Supplementary Tables 1 and 2 for exact tests and significance values). In the case of the KCl buffers, the addition of lidocaine led to decreased cell viability at lower pulse energy applications, but the statistical significance of the viability response was not affected. This suggests that intracellular stores of Mg^2+^ allow for cell recovery at lower energy pulse applications. A more dramatic effect was observed with the addition of lidocaine to the MgCl_2_ buffer. In this case, upon surpassing an applied energy threshold, a dramatic decrease in the viability response curve was observed, resembling the viability response of the KCl -based buffer solution. This data provides some evidence that ATPase activation, through binding of extraneous Mg^2+^, is necessary to conserve cell viability following high-energy electroporation pulse applications, enhancing the cell population’s electrical energy tolerance to result in reversible electroporation.

### Electroporation outcome score

**Figure 4:**
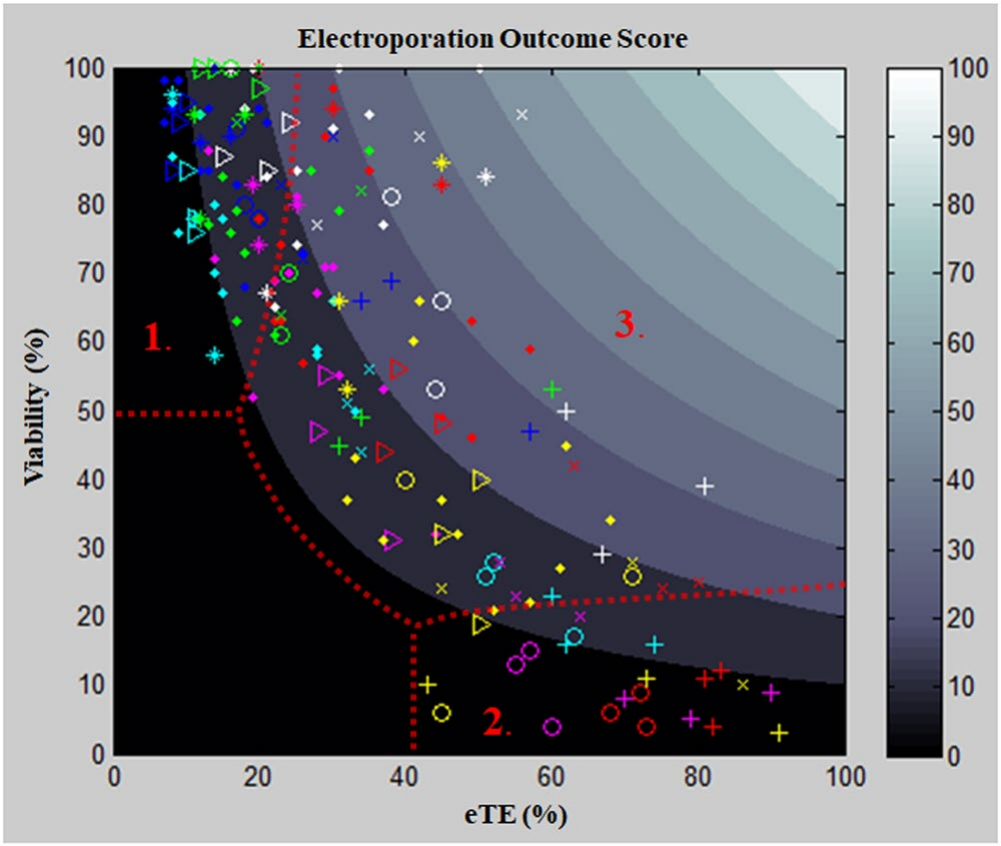
Electroporation outcome score. Buffer color code: blue—MgCl_2_ (500 μS/cm), red—KCl (500 μS/cm), cyan— MgCl_2_ (2000 μS/cm), magenta— KCl (2000 μS/cm), white— MgCl_2_/KCl (500 μS/cm) green—MgSO_4_ (500 μS/cm), yellow—KCl with trehalose (500 μS/cm). Pulse application code: *—control pulse (1.2 kV/cm: 1 ms), ◊—constant applied energy, Δ—1.8 kV/cm: 670 μs, **×**—2.4 kV/cm: 500 μs, ○—3.6 kV/cm: 330 μs, **+**—4.8 kV/cm: 250 μs. Region 1 is representative of high viability with low eTE and is comprised of Mg^2+^ (+) buffers and/or low energy pulse applications. Region 2 is representative of low viability with high eTE and is comprised of Mg^2+^ (−) and/or high energy pulse applications. Region 3 is moderate to high viability with moderate eTE and is comprised of Mg^2+^ (−) at low energy pulse applications and Mg^2+^ (+) at higher energy pulse applications, with the Mg^2+^/K^+^-buffer resulting in the best outcome scores.

To explore the combined effects of both viability and eTE on electroporation outcomes, where one seeks to obtain both high viability and high transfection efficiency, a scoring metric was created (Fig. 4). This metric is the product of the cell viability (% living cells) and transfection efficiency percentages (of those living cells, % transfected/expressing GFP), with scores ranging from 0 a.u. to 100 a.u., for each experimental condition as shown in Fig. 4. This metric allows for the discrimination between both the different buffer solutions and pulse applications in the study. In our results we find the existence of three distinct regions: high viability with low eTE (1), low viability with high eTE (2), and moderate-to-high viability with moderate eTE (3). The high viability with low eTE group (1) is composed of all buffer solutions at lower-energy pulse applications and Mg^2+^-containing buffers at higher-energy pulse applications. In this region it is likely that few cells were permeabilized, resulting in low transfection outcomes. The low viability/high eTE group (2) is composed of buffers lacking Mg^2+^ at high-energy pulse applications. Therefore, cells suspended in a KCl-based buffer that remain viable following high-energy pulses are likely to be successfully transfected. Cells in this group are presumably over-permeabilized and unable to recover, resulting in lower viability outcomes. The final group, which we consider optimal according to our metric, is the moderate to high viability/moderate eTE (3). This region of outcomes is the result of most cells undergoing reversible electroporation, up-taking and transcribing the pDNA, while surviving the overall electroporation process. This group mainly consists of the KCl buffer at 500 μS/cm with low- to moderate- energy pulse applications and the MgCl_2_/KCl mixture buffer at 500 μS/cm for all pulse applications, with the mixture buffer resulting in the highest outcomes scores. These data further show the benefits of including an optimal amount of Mg^2+^ in the electroporation buffer. The highest electroporation outcome was 52 a.u. (MgCl_2_/KCl, 2.4 kV/cm: 500 μs, viability—93%, eTE—56%), demonstrating substantial room for improvement, particularly in eTE. Recent works have demonstrated and noted the important role of endocytotic pathways in successful electro-transfection outcomes, specifically the inhibitory effect of prolonged cold temperatures on cells following electroporation pulse application^26,36–38^. Taking this into consideration, by eliminating the brief ice incubation period following electroporation, an enhancement in the electroporation outcomes could be expected. Nevertheless, these results provide important insights and a methodology to show a quantifiable comparison between the different effects the chosen buffer compositions have on electroporation outcomes and thereby allow for the rational development of electroporation buffer composition. In particular, the results highlight the need to optimize the Mg^2+^ ion concentration to enhance both cell viability and eTE outcomes, which is in agreement with other reports^26^. Utilizing these findings in tandem with optimization of the other variables will increase electroporation-outcome scores, leading to further adoption of electroporation as a modality to perform clinically-relevant cell transfections.

### Future work

We anticipate that the approach used in designing these studies can be expanded to include other buffer compositions and cell types (i.e., PBMCs, Jurkat cells, HEK293T cells, etc.). This will help inform the development of optimized electroporation protocols for specific applications such as gene editing, CAR T-cell generation, etc. However, each electroporation buffer (i.e., cell-culture media, phosphate-buffered saline, and other phosphate-based buffers) and cell type may yield different viability and eTE for a given application and must be considered moving forward. We also wish to further examine our hypothesis that the effect of magnesium on post electroporation viability and eTE is due to magnesium’s role as a co-factor in biochemical processes. We believe the systematic modification of buffer composition coupled with keeping pulse energy and total charge flux constant represents an improved approach to determining optimized electroporation protocols in such a large experimental parameter space.

## Conclusion

In this work we showcase the effect that different compositions of electroporation buffer have on cell viability and eTE. Most notably, the results confirm the important role that Mg^2+^ plays as an enzymatic co-factor leading to an enhancement of cell viability while hindering eTE following the electroporation process. Electroporation outcomes were compared using a quantifiable metric, the product of the cell viability and eTE percentages, allowing for discrimination between experimental results. These results suggest that an optimal concentration of Mg^2+^ should be included within the electroporation-buffer solution to strengthen a cell population’s ability to undergo reversible electroporation.

## Supplementary information

Supplementary File.

## Data Availability

The datasets generated during and/or analyzed during the current study can be found in the Figures, Tables and Supplementary information or are available upon request.
