# Supplementary material for: The effects of electroporation buffer composition on cell viability and electro-transfection efficiency
Source: Sci Rep. 2020 Feb 20;10:3053. doi: 10.1038/s41598-020-59790-x (PMC7033148; doi:10.1038/s41598-020-59790-x)
Supplement: Supplementary file 1 — Supplementary File. [file 41598_2020_59790_MOESM1_ESM.docx]

**The effects of electroporation buffer composition on cell viability and electro-transfection efficiency**

**Joseph J. Sherba^1^, Stephen Hogquist^1^, Hao Lin^2^, Jerry W. Shan^2^, David I. Shreiber^1^, Jeffrey D. Zahn^1,*^**

^1^ Rutgers, The State University of New Jersey, Department of Biomedical Engineering, Piscataway, 08854, United States.

^2^ Rutgers, The State University of New Jersey, Department of Mechanical and Aerospace Engineering, Piscataway, 08854, United States.

^*^Jeffrey D. Zahn, [jdzahn@soe.rutgers.edu](mailto:jdzahn@soe.rutgers.edu)

This supplementary file contains the results from all of the statistical analyses performed, the two-way ANOVAs and post hoc analysis. These are found in Tables 1 and 2, respectively. Table 1 is further separated into 3 sections, containing the results for the constant applied energy pulse applications, constant charge flux pulse applications, and ATPase inhibition, respectively. Each analysis reports the resulting statistical significance values from each two-way ANOVA. Similarly, Table 2 is separated into three groups and reports the results from the post-hoc analyses that reached statistical significance for the constant charge flux pulse applications. These groupings are: viability, electro-transfection efficiency, and ATPase inhibition viability. Figure 1 is the viability data plots for the various buffer compositions for the pulse applications of the same energy. This demonstrates that there are no significant changes in viability when the same pulse energy is applied. However, at the highest charge flux applications, a slight decrease in cell viability for most electroporation buffers is observed. Lastly, Figure 2 is a plot of viability versus applied pulse energy for the 500 μS/cm Mg^2+^ -containing buffer compositions. No significant differences are found at any pulse energy, indicating the enhancement of viability is independent of the Mg^2+^ source.

**Supplementary Figures:**

| **Buffer Composition** | **Charge Flux** | **Interaction** |
| --- | --- | --- |
| **Constant Applied Energy --Viability** | | |
| **All Buffers (500 μS/cm)** | | |
| n.s. | n.s. | n.s |
| **All Buffers (2000 μS/cm)** | | |
| n.s. | *p* = 0.0044 | n.s. |
| **Constant Applied Energy --eTE** | | |
| **All Buffers (500 μS/cm)** | | |
| *p* < 0.0001 | *p* = 0.0073 | n.s. |
| **Mg^2+^ -Containing Buffers (500 μS/cm)** | | |
| *p* < 0.0001 | n.s. | n.s. |
| **All Buffers (2000 μS/cm)** | | |
| *p* < 0.0001 | *p* < 0.0001 | n.s. |

**Supplementary Table 1.1. Two-way ANOVA results for constant applied energy pulsing conditions—Viability and eTE.** n.s.- not significant.

| **Buffer Composition** | **Appl. Energy** | **Interaction** |
| --- | --- | --- |
| **Constant Charge Flux--Viability** | | |
| **All Buffers (500 μS/cm)** | | |
| *p* < 0.0001 | *p* < 0.0001 | *p* < 0.0001 |
| **Mg^2+^ -Containing Buffers (500 μS/cm)** | | |
| n.s. | *p* < 0.0001 | n.s. |
| **All Buffers (2000 μS/cm)** | | |
| *p* < 0.0001 | *p* < 0.0001 | n.s. |
| **Constant Charge Flux--eTE** | | |
| **All Buffers (500 μS/cm)** | | |
| *p* < 0.0001 | *p* < 0.0001 | *p =* 0.0148 |
| **Mg^2+^ -Containing Buffers (500 μS/cm)** | | |
| *p* < 0.0001 | *p* < 0.0001 | n.s. |
| **All Buffers (2000 μS/cm)** | | |
| *p* < 0.0001 | *p* < 0.0001 | n.s. |

**Supplementary Table 1.2. Two-way ANOVA results for constant charge flux pulsing conditions—Viability and eTE.** n.s.- not significant.

| **Buffer Composition** | **Appl. Energy** | **Interaction** |
| --- | --- | --- |
| **ATPase Inhibition--Viability** | | |
| **MgCl_2_ versus MgCl_2_ with Lidocaine** | | |
| *p* < 0.0001 | *p* < 0.0001 | *p* < 0.0001 |
| **KCl versus KCl with Lidocaine** | | |
| *p* < 0.0001 | *p =* 0.0074 | n.s. |
| **KCl versus MgCl_2_ with Lidocaine** | | |
| *p* < 0.0001 | *p* < 0.0001 | n.s. |

**Supplementary Table 1.3. Two-way ANOVA results for ATPase inhibition experiments.** n.s.- not significant.

| **Buffer 1** | **Buffer 2** | **p-value** |
| --- | --- | --- |
| **Constant Charge Flux--Viability** | | |
| **1.2 kV/cm : 1 ms** | | |
| MgCl_2_ (500) | KCl (Trehalose) | <0.0001 |
| KCl (500) | KCl (Trehalose) | <0.0001 |
| MgCl_2_/KCl | KCl (Trehalose) | <0.0001 |
| MgSO_4_ | KCl (Trehalose) | <0.0001 |
| **1.8 kV/cm : 670 μs** | | |
| MgCl_2_ (500) | KCl (500) | 0.0097 |
| MgCl_2_ (500) | KCl (Trehalose) | <0.0001 |
| MgCl_2_/KCl | KCl (500) | 0.02 |
| MgCl_2_/KCl | KCl (Trehalose) | 0.0001 |
| MgSO_4_ | KCl (500) | 0.0005 |
| MgSO_4_ | KCl (Trehalose) | <0.0001 |
| MgCl_2_ (2000) | KCl (2000) | 0.0051 |
| **2.4 kV/cm : 500 μs** | | |
| MgCl_2_ (500) | KCl (500) | 0.0006 |
| MgCl_2_ (500) | KCl (Trehalose) | <0.0001 |
| MgCl_2_/KCl | KCl (500) | 0.0001 |
| MgCl_2_/KCl | KCl (Trehalose) | <0.0001 |
| MgSO_4_ | KCl (500) | 0.0015 |
| MgSO_4_ | KCl (Trehalose) | 0.0001 |
| **3.6 kV/cm : 330 μs** | | |
| MgCl_2_ (500) | KCl (500) | <0.0001 |
| MgCl_2_ (500) | KCl (Trehalose) | <0.0001 |
| MgCl_2_/KCl | KCl (500) | <0.0001 |
| MgCl_2_/KCl | KCl (Trehalose) | 0.0076 |
| MgSO_4_ | KCl (500) | <0.0001 |
| MgSO_4_ | KCl (Trehalose) | 0.0002 |
| **4.8 kV/cm : 250 μs** | | |
| MgCl_2_ (500) | KCl (500) | 0.0006 |
| MgCl_2_ (500) | KCl (Trehalose) | 0.0005 |
| MgSO_4_ | KCl (500) | 0.0158 |
| MgSO_4_ | KCl (Trehalose) | 0.0125 |

**Supplementary Table 2.1. Statistical significance achieved in constant charge flux conditions—viability.** Numbers in parenthesis indicate buffer conductivity (μS/cm). KCl (Trehalose), MgCl_2_/KCl, and MgSO_4_ buffers had a final conductivity of 500 μS/cm.

| **Buffer 1** | **Buffer 2** | **p-value** |
| --- | --- | --- |
| **Constant Charge Flux--eTE** | | |
| **1.2 kV/cm : 1 ms** | | |
| MgCl_2_ (500) | KCl (500) | <0.0001 |
| MgCl_2_ (500) | MgCl_2_/KCl | 0.0004 |
| MgCl_2_ (500) | KCl (Trehalose) | <0.0001 |
| MgCl_2_/KCl | MgSO_4_ | 0.0342 |
| MgCl_2_/KCl | KCl (Trehalose) | 0.0004 |
| MgSO_4_ | KCl (500) | 0.0012 |
| MgSO_4_ | KCl (Trehalose) | <0.0001 |
| KCl (500) | KCl (Trehalose) | 0.016 |
| MgCl_2_ (2000) | KCl (2000) | 0.0196 |
| **1.8 kV/cm : 670 μs** | | |
| MgCl_2_ (500) | KCl (500) | 0.0033 |
| MgCl_2_ (500) | KCl (Trehalose) | <0.0001 |
| MgCl_2_/KCl | KCl (Trehalose) | 0.0105 |
| MgSO_4_ | KCl (500) | 0.0299 |
| MgSO_4_ | KCl (Trehalose) | 0.0015 |
| MgCl_2_ (2000) | KCl (2000) | 0.008 |
| **2.4 kV/cm : 500 μs** | | |
| MgCl_2_ (500) | KCl (500) | <0.0001 |
| MgCl_2_ (500) | KCl (Trehalose) | <0.0001 |
| MgCl_2_/KCl | KCl (500) | 0.0033 |
| MgCl_2_/KCl | KCl (Trehalose) | 0.0299 |
| MgSO_4_ | KCl (500) | <0.0001 |
| MgSO_4_ | KCl (Trehalose) | 0.0001 |
| MgCl_2_ (2000) | KCl (2000) | 0.0031 |
| **3.6 kV/cm : 330 μs** | | |
| MgCl_2_ (500) | KCl (500) | <0.0001 |
| MgCl_2_ (500) | MgCl_2_/KCl | 0.0413 |
| MgCl_2_ (500) | KCl (Trehalose) | 0.001 |
| MgCl_2_/KCl | KCl (500) | 0.0072 |
| MgSO_4_ | KCl (500) | <0.0001 |
| MgSO_4_ | KCl (Trehalose) | 0.0033 |
| **4.8 kV/cm : 250 μs** | | |
| MgCl_2_ (500) | KCl (500) | <0.0001 |
| MgCl_2_ (500) | MgCl_2_/KCl | 0.0151 |
| MgCl_2_ (500) | KCl (Trehalose) | 0.0214 |
| MgCl_2_/KCl | MgSO_4_ | 0.0105 |
| MgSO_4_ | KCl (500) | <0.0001 |
| MgSO_4_ | KCl (Trehalose) | 0.0151 |

**Supplementary Table 2.2. Statistical significance achieved in constant charge flux conditions—eTE.** Numbers in parenthesis indicate buffer conductivity (μS/cm). KCl (Trehalose), MgCl_2_/KCl, and MgSO_4_ buffers had a final conductivity of 500 μS/cm.

| **Buffer 1** | **Buffer 2** | **p-value** |
| --- | --- | --- |
| **ATPase Inhibition--Viability** | | |
| **1.2 kV/cm : 1 ms** | | |
| MgCl_2_ (500) | KCl (500) | 0.0152 |
| MgCl_2_ (500) | KCl (w/ Lidocaine) | <0.0001 |
| KCl (500) | MgCl_2_ (w/ Lidocaine) | 0.0328 |
| KCl (500) | KCl (w/ Lidocaine) | 0.0235 |
| MgCl_2_ (w/ Lidocaine) | KCl (w/ Lidocaine) | 0.0002 |
| **1.8 kV/cm : 670 μs** | | |
| MgCl_2_ (500) | KCl (500) | 0.0006 |
| MgCl_2_ (500) | KCl (w/ Lidocaine) | <0.0001 |
| KCl (500) | MgCl_2_ (w/ Lidocaine) | 0.0021 |
| KCl (500) | KCl (w/ Lidocaine) | 0.0465 |
| MgCl_2_ (w/ Lidocaine) | KCl (w/ Lidocaine) | <0.0001 |
| **2.4 kV/cm : 500 μs** | | |
| MgCl_2_ (500) | KCl (500) | <0.0001 |
| MgCl_2_ (500) | KCl (w/ Lidocaine) | <0.0001 |
| KCl (500) | MgCl_2_ (w/ Lidocaine) | 0.0094 |
| MgCl_2_ (w/ Lidocaine) | KCl (w/ Lidocaine) | <0.0001 |
| **3.6 kV/cm : 330 μs** | | |
| MgCl_2_ (500) | KCl (500) | <0.0001 |
| MgCl_2_ (500) | MgCl_2_ (w/ Lidocaine) | <0.0001 |
| MgCl_2_ (500) | KCl (w/ Lidocaine) | <0.0001 |
| **4.8 kV/cm : 250 μs** | | |
| MgCl_2_ (500) | KCl (500) | <0.0001 |
| MgCl_2_ (500) | MgCl_2_ (w/ Lidocaine) | <0.0001 |
| MgCl_2_ (500) | KCl (w/ Lidocaine) | <0.0001 |

**Supplementary Table 2.3. Statistical significance achieved in ATPase inhibition conditions—viability.** All buffers had a final conductivity of 500 μS/cm and sucrose was used as the osmotic balancing agent.


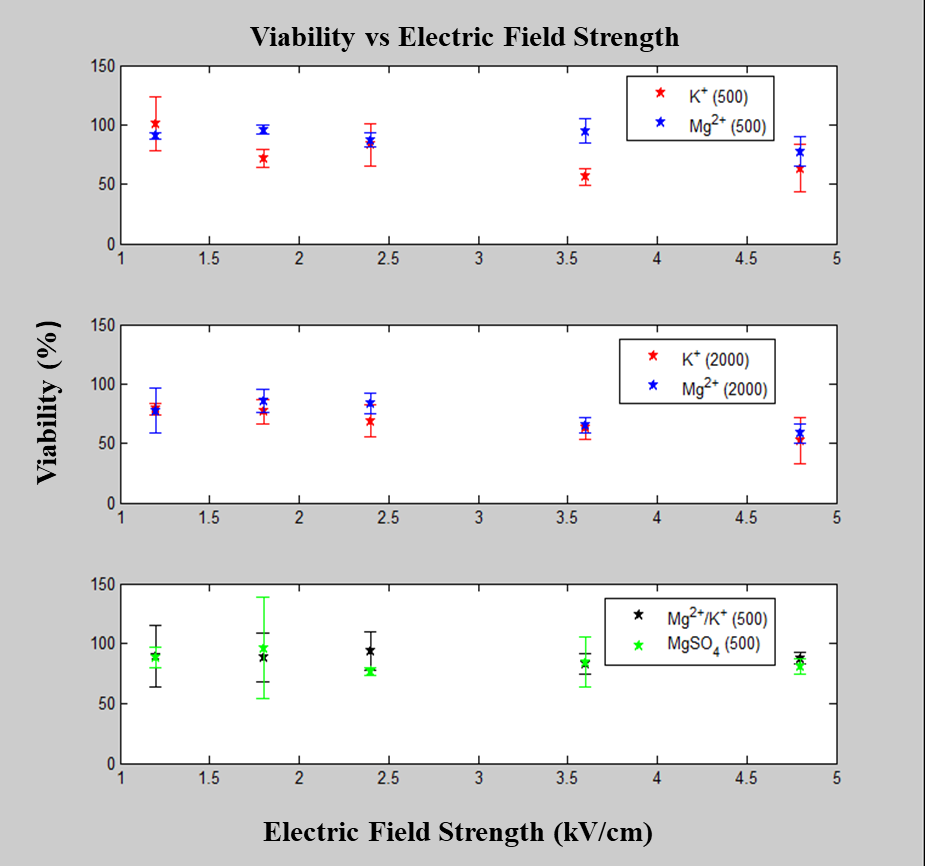


**Supplementary Figure 1. Viability vs electric field strength for constant applied energy.** (Top) KCl and MgCl_2_ buffers at 500 μS/cm. (Middle) KCl and MgCl_2_ buffers at 2000 μS/cm. (Bottom) MgCl_2_/KCl mixture and MgSO_4_ buffers at 500 μS/cm. A slight decrease in viability is observed at high electric field strength, short duration, pulse applications.


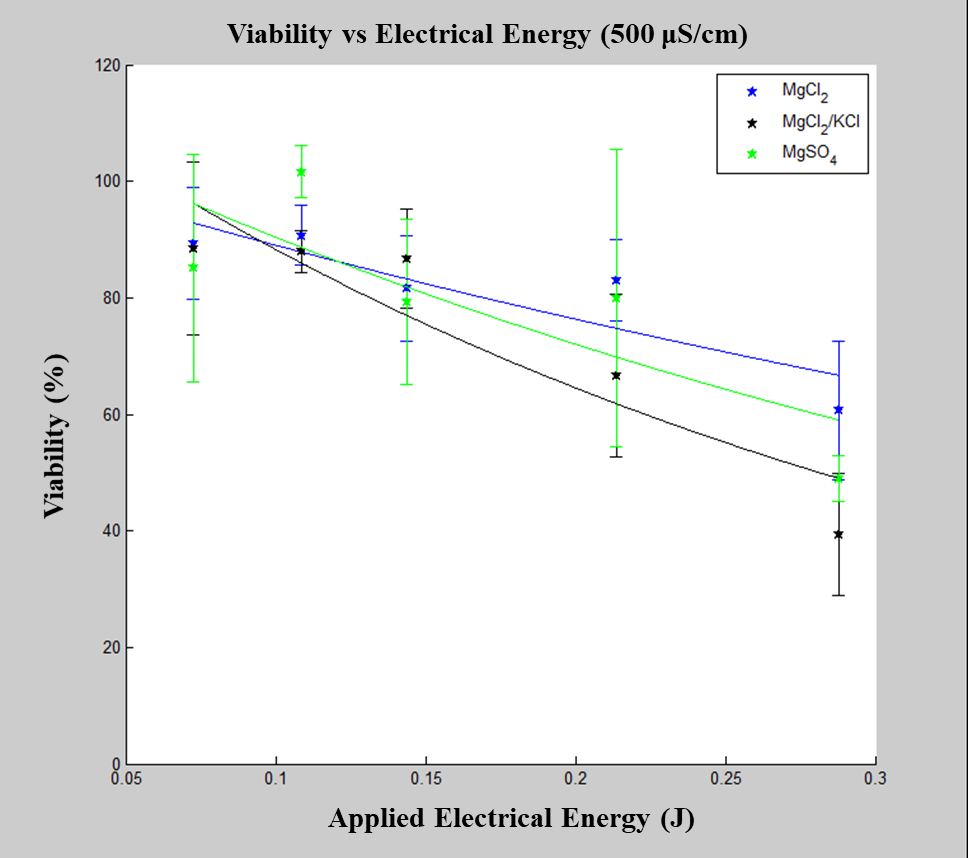


**Supplementary Figure 2. Viability vs applied electrical energy for Mg^2+^ containing buffer solutions.** Each buffer with a final conductivity of 500 μS/cm and sucrose as the osmotic balancing agent. Statistical significance (*p* < 0.05) was not reached for any pulse applications when comparing MgCl_2_, MgSO_4_, and MgCl_2_/KCl mixture to each other. Data fit to a two-term exponential.
